# Subacute uterine inversion following an induced abortion in a teenage girl: a case report

**DOI:** 10.1186/s12905-020-01089-0

**Published:** 2020-10-02

**Authors:** Asiphas Owaraganise, Leevan Tibaijuka, Joseph Ngonzi

**Affiliations:** Department of Obstetrics and Gynecology, Mbarara University of Science and Technology, Mbarara, Uganda

**Keywords:** Case report, Subacute, Uterine inversion, Unsafe abortion, Johnson’s manoeuvre, Hysterectomy

## Abstract

**Background:**

Subacute uterine inversion is a very rare complication of mid-trimester termination of pregnancy that should be considered in a situation where unsafe abortion occurs.

**Case presentation:**

We present a case of subacute uterine inversion complicated by hypovolemic shock following an unsafe abortion in a 17-year-old nulliparous unmarried girl. She presented with a history of collapse, mass protruding per vagina that followed Valsalva, and persistent lower abdominal pain but not vaginal bleeding. This followed her second attempt to secretly induce an abortion at 18 weeks amenorrhea. On examination, she was agitated, severely pale, cold on palpation, with an axillary temperature of 35.8 °C, a tachycardia of 143 beats per minute and unrecordable low blood pressure. The abdomen was soft and non-tender with no palpable masses; the uterine fundus was absent at its expected periumbilical position and cupping was felt instead. A fleshy mass with gangrenous patches protruding in the introitus was palpated with no cervical lip felt around it. We made a clinical diagnosis of subacute uterine inversion complicated with hypovolemic shock and initiated urgent resuscitation with crystalloid and blood transfusion. Non-operative reversal of the inversion failed. Surgery was done to correct the inversion followed by total abdominal hysterectomy due to uterine gangrene.

**Conclusion:**

Our case highlights an unusual presentation of subacute uterine inversion following unsafe abortion. This case was managed successfully but resulted in significant and permanent morbidity.

## Background

Subacute puerperal uterine inversion occurs when the uterine fundus collapses into the endometrial cavity between 24 h to 1 month postpartum [1]. It is a rare gynaecological emergency in practice and literature [2] but potentially fatal when not diagnosed and treated fast. Uterine inversion is an unpredictable obstetric emergency that should be considered when a woman in puerperium presents with postpartum bleeding, low blood pressure, abdominal pain and a mass in the vagina. Its management involves anatomical repositioning of the uterus while preventing re-inversion, and treating haemorrhage while restoring the patient’s haemodynamic stability. Urgent resuscitation with crystalloids and blood transfusion improves patient outcomes [3, 4]. Reversing uterine inversion is achieved using non-operative or surgical means. Non-surgical options include manual replacement and hydrostatic reduction while surgical correction includes laparotomy plus Huntington’s procedure alone or combined with incision of the cervical constriction ring. The time interval between its recognition and correction alongside resuscitation measures instituted are the major drivers of the prognosis [5]. We describe below a maternal near-miss case we managed successfully in our department followed by a brief discussion of the same.

## Case presentation

A 17-year-old nulliparous unmarried girl was carried into the admission room at Mbarara regional referral hospital’s gynaecology ward. The chief complaints were: collapse, mass protruding per vagina that followed Valsalva (defecation), and persistent lower abdominal pain but without vaginal bleeding. She had secretly induced abortion at 18 weeks amenorrhea with misoprostol at a rural clinic. Products of conception were not expelled within 4 days prompting her to seek surgical uterine evacuation at a different clinic located ~ 40 km away. The third day after surgical evacuation, she collapsed at home and was brought to our facility. The pregnancy was unplanned. Contraception had been desired but was not readily available.

On examination, she was agitated, severely pale, and cold (temperature 35.8 °C), in a state of shock with a feeble radial pulse at 143 beats/minute, and nonrecordable low blood pressure. The abdomen was soft and non-tender with no palpable masses. On vaginal inspection, a fleshy mass with gangrenous patches measuring about 8 cm by 5 cm was protruding in the introitus. The raw surface was not actively bleeding but foul-smelling pus oozed out of the necrotic patches of the inverted uterine fundus. On digital vaginal examination, the cervical lip was not felt around the mass, on bimanual palpation, the uterine fundus was absent at its expected periumbilical position and cupping was felt. A clinical diagnosis of subacute uterine inversion with hemorrhagic shock was made.

We called for immediate assistance, informed anaesthesiology staff, and blood bank personnel. Simultaneously, two large-bore intravenous lines plus crystalloid infusion and blood transfusion were initiated. Three units of whole blood were transfused. The antibiotics given were intravenous ceftriaxone and metronidazole while the analgesic was an injection of diclofenac. The pre-transfusion complete blood count showed severe anaemia with a haemoglobin concentration of 4.5 g/dl, and 12,600 leucocytes per microliter with 89% neutrophilia; other parameters were within normal ranges.

She was then transferred urgently to the operation theatre for examination under anaesthesia, and the repositioning of the uterus. Nonsurgical correction using Johnson’s and O Sullivan’s methods were attempted with no success. We proceeded and performed a laparotomy. Intraabdominal, the classic flowerpot appearance was seen as the cupping of the uterus, along with tubes, ovaries, round, and ovarian ligaments. Both tubes were found congested and thickened. We attempted Huntington’s procedure to correct inversion in vain. The pouch of Douglas was markedly oedematous and friable. Anterior incision (Ocejo incision) was made at the anterior surface of the uterus after reflecting bladder peritoneum, transected the constriction ring. The inverted uterus was then manually reduced. The fundus was necrotic and a total abdominal hysterectomy performed. The postoperative period was uneventful, Table 1.

**Table 1:** Subacute uterine inversion case management timeline

Preceding events	The nulliparous unmarried adolescent had not planned to become pregnant, and lacked access to safe contraceptive. She attempted to secretly induce abortion from rural clinic without success. She had surgical uterine evacuation at a distant clinic and suffered serious complications		
Dates	Visit(s) summary	Investigations done	Treatment given
**26.01.2020**	Adolescent sought abortion service at rural clinic but did not succeed.	Not documented	•Oral misoprostol
**30.01.2020**	Sought further assistance at another peripheral clinic	Not documented	•Surgical uterine evacuation was conducted patient discharged
**02.02.2020**	Initial visit: Clinical diagnosis of Subacute uterine inversion complicated with hemorrhagic shock and sepsis was made	Full hemogram showed hemoglobin level of 4.5 g/dl, and 12,600 leucocytes per microliter predominantly neutrophils (89%)	•Immediate resuscitation with crystalloid and blood transfusion (3 units). •Initiated injectable diclofenac 75 mg, metronidazole 500 mg and ceftriaxone 2 g
**03.02.2020 to 08.02.2020**	Inpatient care: Patient generally had uneventful post-operative period.	N/A	•Urgent life-saving surgical correction of the inversion, and total abdominal hysterectomy due to necrotic uterine fundus •Continued fluid and blood resuscitation (1 unit) as well as injectable antibiotics and analgesia •Had a psychotherapist review
**09.02.2020**	Discharge day	Post transfusion hemoglobin level was 8.1 g/dl	•Discharged on oral ferrous 200 mg and cefixime 500 mg daily for five days •Given appointment of 21.02.2020
**25.02.2020**	Follow up visit	•Came three days past the appointment date •She had well recovered, and wound healed •Hemoglobin level was 9.7 g/dl •Had concern about future fertility options	Linked to hospital’s social worker for psychosocial support and community liaison

## Discussion and conclusions

Uterine inversion occurs when the uterine fundus collapses into the endometrial cavity, turning the uterus partially or completely inside out. The incidence of puerperal uterine inversion ranges from 1 in 3500 to 20,000 deliveries [5, 6]. It is a rare complication of abortion, but when it occurs, it is a life-threatening gynaecological emergency [7]. If the uterine inversion is not timely recognized and reduced, the resulting severe haemorrhage and shock can lead to maternal death [8]. Our case presented with a life-threatening haemorrhagic shock and suffered serious morbidity—removal of the uterus.

Uterine inversion is classified according to the extent of inversion and timing of occurrence. Four categories are recognised by the extent of inversion. They include: first-degree also called incomplete (fundus remains within the endometrial cavity); second-degree also called complete (fundus protrudes through cervical os); third-degree also called prolapsed (fundus protrudes to or beyond the introitus); and then fourth-degree also called total (both the uterus and vagina are inverted. Majority of cases present as complete inversion [5, 9]. Our patient presented with the fundus protruding beyond introitus but the vagina was not inverted. Categorisation according to the time of occurrence can either be puerperal (more common) or nonpuerperal (commonly associated with tumours). Puerperal inversion is further sub-grouped into acute—within 24 h postpartum, subacute—24 h up to 1 month, and chronic—above 1 month postpartum [10]. Our patient presented with subacute inversion on the 3rd day post-abortion.

The pathogenesis of uterine inversion has been attributed to the use of excessive cord traction and fundal pressure (Credé manoeuvre) or use of uterine relaxants [9]. Also, at 18 weeks gestation, the placenta is firmly attached to the uterus [11]. We think our patient meets both conditions. She presented at 18 gestation weeks with a history of two attempts to evacuate the uterus at different rural clinics.

The extent and time of occurrence of the inversion determine the clinical picture of the patient at presentation. Usually patient present with vaginal bleeding, lower abdominal pain, smooth, round mass protruding from the cervix or vagina, and urinary retention. Commonly uterine inversion presents as severe postpartum haemorrhage and hypovolemic shock [2] The patient presented with protruding mass and hypovolemic shock. Diagnosis is frequently clinical as was in our case but, ultrasound examination shows abnormal uterine fundal contour with a homogeneous globular mass within the uterus. Differential diagnosis includes prolapsed fibroid, cervical polyp and Nabothian cyst. Advanced radiology workup like Magnetic Resonance Imaging is seldom required [12].

Usually, the goals of management of puerperal uterine inversion are to reduce the inversion, treat the haemorrhage and shock that may occur, and prevent recurrent inversion. This requires that the initial intervention processes to manage acute uterine inversion should be performed simultaneously. When such measures to replace the uterus fail, surgical correction of the inversion should be done at laparotomy. We alternately employed two nonsurgical correction techniques: initially used Johnson’s and later O Sullivan’s method but still failed to correct the inversion.

Johnson’s manoeuvre is commonly used to manually reverse uterine inversion with the chances of immediate reduction varying in the range 43–88% [13]. The procedure consists of hand-pushing of the inverted fundus through the cervical ring. The uterine fundus is held in the palm of the dominant hand and the tips of the fingers kept at the utero-cervical junction. The entire hand plus part of the forearm is placed inside the vagina and pressure directed along the axis of the vagina towards the umbilicus so as to raise the fundus above the level of the umbilicus. The tension generated relaxes and widens the cervical ring which facilitates passage of the fundus through the ring. The other hand supports the uterus at the anterior abdomen [4, 14] Placental removal is advised after repositioning of the uterus to avoid shock and excessive haemorrhage; however, an attached placenta increases the bulk and makes manual replacement difficult [13]

Alternatively, a non-surgical reversal of uterine inversion can be achieved by hydrostatic reduction—the O’Sullivan technique. The procedure is performed in the operating theatre with the woman in Trendelenburg lithotomy position. Uterine relaxants, such as glyceryl trinitrate, magnesium sulphate and terbutaline, may be used to augment the reversal process [15]. The externalised uterus with/out attached placenta is replaced into the vagina. A suction cap such as neonatal ventilation mask is placed inside the vagina to form a seal with the vaginal walls. The assistant’s hand may be applied to block the introitus, however, maintaining a tight seal remains challenging. A bag containing 3–5 l of warm sterile water or isotonic saline solution is then hung 100–150 cm above vaginal level and connected through a rubber hose to the nozzle placed in the posterior fornix. The accumulating free-flowing fluid generates hydrostatic pressure that gradually distends vagina, relieves cervical constriction, and pushes the fundus upwards into its natural position [16, 17].

If manual replacement and hydrostatic reduction fail to reduce uterine inversion, laparotomy is warranted. Intraabdominal, Huntington’s technique is used to correct the inversion. The procedure involves exposing the inversion site which typically appears cup-like in place of the uterus with bilateral fallopian tubes, ovaries and round ligaments retracted inwards. The uterine fundus is gently retracted superiorly using two atraumatic instruments like Babcock clamp or Allis forceps grasping the round ligaments and moving them by adjacent alternate intervals medially. By doing this the uterus is pulled out of the constriction ring and restored to its normal position. Sometimes, simultaneous reduction may also be attempted vaginally to facilitate correction of the inversion [3, 18].

However, Huntington’s techniques may fail to reverse the inversion. In this situation, the surgeon first incises the cervical ring structure along the vertical plane either anteriorly—Ocejo incision or posteriorly—Haultain’s technique [3]. Incising the cervical ring structure creates additional space for manual replacement of the uterus or completing Huntington’s procedure. Repair of vagina, cervix, and uterus is done using interrupted absorbable suture once the inversion is reversed. Haultain’s technique is preferred over Ocejo incision owing to less risk of bladder injury [19]. In our case, intraabdominal, Huntington’s correction failed and we first excised the anterior constriction ring to correct the inversion. Marked tissue inflammation in the rectouterine pouch made it impossible to use the preferred posterior approach.

To prevent re-inversion, an oxytocic is added to firmly hold the fundus in position. The Matsubara-Yano compression suture may be placed to prevent re-inversion and enhance haemostasis [20]. In case of unsuccessful surgical correction, a lifesaving hysterectomy may be done [13]. We performed a hysterectomy in our patient because the uterine fundus was gangrenous and very friable. Standard of care antibiotic, fluid and analgesic therapy was given during the post-operative period. Although not commonly used, laparoscopic-assisted reduction of uterine inversion has been reported [21].

Our case report suggests that subacute uterine inversion can be a life-threatening gynaecological emergency following induced unsafe abortion. Given the unpredictable presentation, clinical care teams should continuously be prepared to timely diagnose and treat subacute uterine inversion to prevent serious complications. The report also highlights the need for targeted interventions to improve contraceptive access and utilization as well as harm reduction from unsafe abortion, especially among vulnerable sexually active adolescent girls.

## Data Availability

Not applicable.
